# Examining the Performance of Fog-Aided, Cloud-Centered IoT in a Real-World Environment

**DOI:** 10.3390/s21216950

**Published:** 2021-10-20

**Authors:** Mohammed A. Aleisa, Abdullah Abuhussein, Faisal S. Alsubaei, Frederick T. Sheldon

**Affiliations:** 1 Department of Computer Science, The University of Idaho, Moscow, ID 83844, USA; sheldon@uidaho.edu; 2 Department of Computer Science, Majmaah University, Al-Majmaah 11952, Saudi Arabia; 3 Information Systems Department, St. Cloud State University, St. Cloud, MN 56301, USA; aabuhussein@stcloudstate.edu; 4 Department of Cybersecurity, University of Jeddah, Jeddah 23890, Saudi Arabia; fsalsubaei@uj.edu.sa

**Keywords:** Internet of Things, performance analysis, fog computing, cloud computing, performance metrics, benchmarking, distributed systems

## Abstract

The fog layer provides substantial benefits in cloud-based IoT applications because it can serve as an aggregation layer and it moves the computation resources nearer to the IoT devices; however, it is important to ensure adequate performance is achieved in such applications, as the devices usually communicate frequently and authenticate with the cloud. This can cause performance and availability issues, which can be dangerous in critical applications such as in the healthcare sector. In this paper, we analyze the efficacy of the fog layer in different architectures in a real-world environment by examining performance metrics for the cloud and fog layers using different numbers of IoT devices. We also implement the fog layer using two methods to determine whether different fog implementation frameworks can affect the performance. The results show that including a fog layer with semi-heavyweight computation capability results in higher capital costs, although the in the long run resources, time, and money are saved. This study can serve as a reference for fundamental fog computing concepts. It can also be used to walk practitioners through different implementation frameworks of fog-aided IoT and to show tradeoffs in order to inform when to use each implementation framework based on one’s objectives.

## 1. Introduction

Cloud computing is an emerging technology that offers high computational power and permanent storage for the Internet of Things (IoT). In cloud-based IoT environments, as the number of IoT devices increases, the amount of data generated from the IoT also increases. This causes high latency due to the long distances between the IoT devices and the cloud. The exchange of data between IoT devices and the cloud increases the utilization of bandwidth and requires increasing resources as the number of IoT devices increases. In addition, operations such as IoT device authentication and authorization, as well as encryption, add computation overheads to the cloud. This means the computation capability must be brought closer to the IoT devices and the resource-demanding tasks must be reserved for the cloud. As such, fog computing emerged to satisfy the demands for frequent computation, communication, and storage by the IoT layer [1,2].

According to the commercial sector, fog computing is a layer of computing that extends the cloud, bringing it closer to the things that generate and process IoT data. Any device with computing, storage, and network connectivity can be a fog node, while fog layer nodes can be deployed anywhere with a network connection (e.g., on top of a traffic light, alongside a railway track [3]). Fog computing involves the following characteristics [1]:

- Low latency: The amount of time it takes for data to send from source to destination is known as network latency. In cloud-based IoT environments, latency is typically high due to the distance between the IoT devices and the cloud. This increases the cloud response time, especially as the number of IoT devices increases, making the cloud unable to support the real-time demands of IoT devices. Fog computing decreases the latency by moving data to the edge of the network and nearer to end users to meet the high processing demands;
- Higher scalability: The ability of a system to manage an increasing quantity of work by adding resources to the system is known as scalability. In cloud-based IoT environments, as the number of IoT devices increases, it becomes challenging for the cloud to accommodate the heavy computation and bandwidth demands of the devices. When the number of IoT devices grows, fog computing can solve this problem by distributing several fog nodes, which can reduce the heavy computation demands on the cloud and provide hierarchal scalability;
- Location awareness: The ability of a device to determine its location, either passively or actively, is referred to as location awareness. This feature is important because it allows applications to provide services better suited to the user and device location, thereby lowering latency;
- Mobility: Computing mobility is the ability to perform computing operations while a connected device is able to move, communicating from any location through a wireless channel. This includes the mobility of IoT nodes as well as fog nodes in cloud-based IoT environments;
- Decentralized architecture: A decentralized network is a network of interconnected devices in which no single entity is the sole authority. Workloads in distributed architectures are distributed among several machines instead of relying on a single central server. This is an important feature of fog computing because applications and services on the fog can process and store data from any end devices, whether it is a fog node or a sensor (i.e., IoT node);
- Heterogeneity: Heterogeneity in networking refers to connecting devices made by different manufacturers running different operating systems using multiple network architectures and protocols. Fog computing heterogeneity is a topological feature that is of particular importance in cloud-based IoT environments, as it enables devices to exchange information, meaning the exchanged information can be used without restrictions;
- Bandwidth optimization: Optimization of the network bandwidth refers to the overall inbound and outbound bandwidth improvements in a network. This allows fog nodes to handle the traffic from billions of devices to prevent congestion and latency problems. This is due to the fact that the massive amounts of data collected by IoT devices may be processed locally rather than being sent to the cloud.

Despite the benefits offered by fog-aided IoT, researchers and practitioners are faced with challenges in the implementation and performance of fog-aided IoT systems.

First, there is a lack of real-life implementation of the many theoretical studies, as highlighted in our earlier work in [1]. Although simulation-based experiments provide easy access to practical results regarding the performance of computing systems, observations and research outcomes may not be generalizable to all scenarios due to the variety of IoT platform providers and device manufacturers; their different implementation frameworks, service specifications, and configurations; and differences in the network architectures and protocols. As such, in order to develop a profound and general insight into the tradeoffs involved in a particular system, it is important to use real IoT platforms built on top of real-world networks (i.e., the Internet) when obtaining analytical results regarding the performance of fog-aided IoT implementation. In addition, it would be interesting to explore the performance differences of fog implementation frameworks interacting with different commercial IoT platforms, such as Amazon IoT and Azure IoT.

Second, due to the diversity of fog-aided IoT environments and the lack of consensus among practitioners and hobbyists on a standard fog computing implementation framework [1], there is a lack of resources showing how to implement efficient fog-aided IoT systems. Most of the implementation frameworks available are either domain-specific, complex, or too abstract to be useful in all scenarios.

Third, although fog computing offers promising solutions to many of the performance and security problems of IoT [4], it also involves various security and privacy risks. For instance, while fog computing is crucial for spreading risks across distributed fog nodes, it also has the untoward effect of increasing the attack surface. This is made worse because fog computing devices interact with devices only; that is, the fog nodes receive IoT data from sensors and send the data to the cloud, and vice versa. This means that no humans are involved in the communication. Although this can be considered an advantage because these interacting devices do not have screens or an on-device user interface, which reduces the attack surface, it can lead to failures or targeted attacks that cannot be easily detected and deterred. Other security and privacy issues in fog-aided IoT also deserve our attention. In this work, we aim to better understand fog-aided IoT environments in order to pave the way for further research to address interesting confidentiality, integrity, and availability violations. This paper makes the following contributions:

- We present two cloud-based IoT environment architectures. The first architecture has a fog layer applied between the IoT devices and the cloud, whereas the second architecture publishes the data directly to the cloud without a fog layer;
- We use two benchmarks to measure the performance of the cloud-based IoT architectures. The first benchmark involves Mosquitto message broker metrics, which are used to measure the performance of the IoT–fog–cloud at the fog computing level. The second benchmark involves AWS message broker metrics, which are used to measure the performance of the two architectures (IoT–cloud and IoT–fog–cloud) to show the impact of the additional middle layer (i.e., the fog layer) on cloud-centered IoT environments;
- We discuss the security and privacy implications of the two architectures presented in this paper, showing what triggers these implications and suggesting methods to address them;
- This study serves as a tutorial reference for fundamental fog computing concepts and aims to walk practitioners through different implementation frameworks for fog-aided IoT and to reveal tradeoffs that inform when to use each implementation based on one’s objectives.

The remainder of this paper is structured as follows. In Section 2, we discuss the background in detail. In Section 3, we present the related work and discuss existing industrial and Message Queuing Telemetry Transport (MQTT) benchmarks. In Section 4, we describe the experiment setups of the two architectures in detail. In Section 5, we provide a description of the AWS IoT metrics and Mosquitto message broker metrics that are used to measure our two architectures. In Section 6, we explain the analysis methods used to perform the experiments on both architectures. In Section 7, we describe the results of the experiments performed on both architectures. In Section 8, we provide an evaluation of the experimental results based on the analysis methods. In Section 9, we present the threats to validity. In Section 10, we provide a discussion and outline the limitations of our work. Finally, we conclude the paper by provided future research directions in Section 11.

## 2. Background

**Figure 1:**
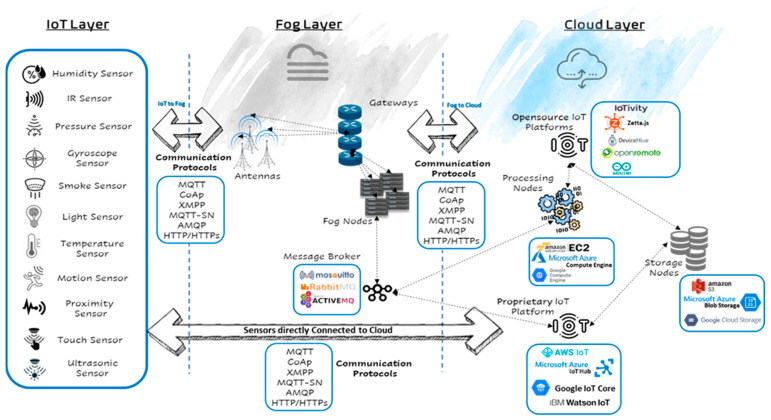
Overview of cloud-aided IoT environments.

Today, IoT sensors are used everywhere and have become crucial in many domains of life. As shown in Figure 1, different kinds of sensors can be found in our homes, cars, workplaces, and other areas, which are sold independently (e.g., smoke sensors, light sensors, temperature sensors, motion sensors, proximity sensors, touch sensors, ultrasonic sensors, humidity sensors, IR sensors, pressure sensors, gyroscope sensors) or as integral parts of sophisticated devices, such as smartphones, which may contain dozens of sensors. These sensors are developed by major manufacturers and are deployed in many sectors, including healthcare, education, communication, transportation, and manufacturing.

Manufacturers have developed IoT platforms to help organizations build fully functional IoT environments. According to AT&T [5], an IoT platform is an end-to-end software framework that pulls together information from sensors, devices, networks, and software, which work together to unlock valuable, actionable data. IoT platforms enable management and automation of connected devices within the IoT universe. There are several proprietary IoT platforms available, including AWS IoT Core [6], Microsoft Azure IoT [7], IBM IoT [8], and Google IoT Core [9], as well as open source IoT platforms such as IoTivity [10], Zetta [11], Arduino IDE [12], DeviceHive [13], and openremote [14]. These IoT platforms usually reside and run on a virtual machine in the cloud that efficiently pulls, processes, and stores the data received from the massive number of IoT sensors.

Classical IoT environments are configured so that IoT sensors are directly connected to the IoT platform and the cloud. In modern architectures, a fog layer is introduced between the IoT sensors and the IoT platform (the cloud) for extra-efficient computation, communication, and storage. Case studies showing the tradeoffs between the two implementation frameworks will be discussed extensively in this paper.

Devices in all layers of IoT environments (see Figure 1) communicate using different protocols. These protocols are listed below [15]:

- MQTT is a lightweight many-to-many communication protocol for IoT that is designed to be a publish–subscribe messaging transport protocol. MQTT is ideal for connecting remote devices with minimal memory consumption and network bandwidth. MQTT is used in a wide variety of domains, such as in industry, health care, and transportation. Port 1883 is the default MQTT port, whereas port 8883 is the default MQTT port over TLS (i.e., secure-mqtt), both of which are registered with the Internet Assigned Numbers Authority (IANA) for Secure MQTT;
- Constrained Application Protocol (CoAp) is a one-to-one User Datagram Protocol (UDP) protocol for transferring state information between the client and server. Despite its ability to preserve resources, CoAP is best-suited to a state transfer model. Since CoAp uses UDP, it does not guarantee the delivery of datagrams. In addition, CoAp is unencrypted. The default CoAP port registered at IANA is 5683;
- Extensible Messaging and Presence Protocol (XMPP) is a secure and near-real-time communication protocol for message-oriented middleware based on XML that enables the exchange of structured but extensible data between any two or more devices over a network. XMPP is mainly used by instant messaging applications such as WhatsApp [16] and Telegram [17]. XMPP offers persistent decentralized connection between devices. As such, no central XMPP servers are needed to communicate; however, to establish a connection between two devices, one of the devices is considered an xmpp-client and communicates over port 5222, while the other is considered an xmpp-server and uses port 5269. XMPP can also use port 5280 for two-way communication. This is called xmpp-bosh, meaning Bidirectional Streams Over Synchronous HTTP (BOSH). XMPP has been used in the literature to network IoT devices, such as in [18,19]. Despite its features and potential, XMPP has some limitations. First, XMPP does not have a Quality of Service (QoS) mechanism. In addition, XMPP streams data in XML format, which introduces overhead due to the text-based communication. These reasons, among others, make MQTT a more popular protocol for IoT, since it has a mechanism for QoS and uses lightweight binary-based communication;
- MQTT For Sensor Networks (MQTT-SN) [20] is considered a modified version of MQTT that is adapted to the attributes of a wireless connection, such as a lossy wireless network. It is designed specifically for wireless sensor networks with scale in mind. MQTT-SN was developed to support non-TCP networks such as UDP. This is another advantage because it makes the communication lighter by eliminating the TCP handshakes;
- The Advanced Message Queuing Protocol [21] (AMQP) is an open standard application layer protocol for middleware. AMQP is designed with more advanced features that introduce more overhead than when using MQTT. These features include message orientation, queuing, routing, reliability, and security. The registered port number for AMQP at IANA is 5672, while for AMQPS (i.e., TLS/SSL encrypted AMQP) it is 5671. For more information on which protocols function best for IoT networks based on the messaging requirements, see [21].

This study focuses on commercial IoT sensors (i.e., DHT11 temperature and humidity sensors) connected using a proprietary IoT platform (i.e., AWS IoT Core). We chose proprietary IoT platforms over the free open source ones because they are popular among industry users due to their faster time to market and lower initial cost.

Amazon Web Services (AWS) provides reliable, scalable, and inexpensive on-demand cloud computing services to individuals, companies, and governments around the world [22]. Customers can benefit from the cloud datacenters distributed in the different locations in many ways, including the low cost (pay as you go) and the massive cloud infrastructure, in order to perform experiments and deploy new applications. AWS offers many services. In this study, we used (1) AWS IoT, (2) Amazon S3, and (3) AWS CloudWatch. AWS IoT allows secure communication and messaging exchange over MQTT for Internet-connected devices such as sensors and micro-controllers in real time [23]. Amazon CloudWatch [24] monitors devices and applications connected to AWS in real time using several metrics [23] to track the connected devices and measure the performance, security, and scalability, among other criteria.

The MQTT protocol is a widely used protocol that is supported by the most platforms and commercial sensors; therefore, we used MQTT as the communication protocol. In addition, we used a Raspberry Pi board to simulate fog nodes for the scenarios in which fog nodes are introduced. To enable the Raspberry Pi boards used in this experiment to communicate over MQTT, we used an MQTT broker software, Eclipse Mosquitto [25]. An MQTT broker is a server that receives all the messages from the IoT devices and publishes them to other devices. MQTT broker also has other benefits, including (1) supporting scalability with many IoT devices, (2) managing credentials and certificates that are used for authentication, (3) decreasing network strain on the cellular network without decreasing security, and (4) excluding the connection of insecure and vulnerable devices. Many MQTT brokers are available, including Eclipse Mosquitto [26], RabbitMQ [26], and ActiveMQ [27]. In this paper, we used Eclipse Mosquitto because it is the most popular and has a lot of resources for implementation. In addition, it is lightweight and suitable for use on all devices, from low-power, single-board computers to full servers. An MQTT broker feature called SYS-Topics [28] is widely used to monitor the Mosquitto MQTT broker by providing metrics about Mosquitto, as well as to track the devices connected to the broker.

In this paper, we present numerical results based on an experiment that uses a real-world IoT platform, sensors, and network (not a simulation) to show the performance tradeoffs of various IoT implementation frameworks and discuss the results. Notably, this experiment in a real environment is vulnerable to real-life cyber or physical attacks, as well as performance failures.

## 3. Related Works

The rapid adoption of cloud-based IoT environments in large scale and with intensive use has induced, among other factors, a growing need to simulate real-life environments to measure the security and performance of the IoT–fog–cloud environments to provide suitable support for the construction of efficient access control models [1]. Benchmarks are one of the ways to measure the security and performance of cloud-based IoT environments. There are several widely used general industry benchmarks that are adopted in many commercial solutions. The Standard Performance Evaluation Corporation (SPEC) provides benchmarks for a wide range of IT components, such as cloud, CPU, storage, power, and virtualization components [29]. The Transaction Processing Performance Council (TPC) also offers a suite of widely used IT industry benchmarks [30]. TPCx-IoT is one of the TPC benchmarks that measures the operating, data storage, and management systems to provide the industry with performance metrics and other available metrics for IoT systems [31,32]. Moreover, HP developed IoTABench, an IoT analytics benchmark for big data scenarios that is used to evaluate the performance and scalability of big data platforms [33]. The benchmark was demonstrated using a smart metering IoT use case and evaluated on the HP Vertica 7 analytics platform, which can handle data for an “electric utility with 40 million smart meters”.

For MQTT, different benchmarks to measure the performance and security of cloud-based IoT environments have been proposed in the literature [34,35]. Two IoT platforms, Things Board and Site Where, have been evaluated using different metrics [36]. In addition, an evaluation of the message transmission processes (i.e., Subscribe and Publish) of the MQTT protocol via wireless and wired clients was presented in [37]. The end-to-end delay and message loss when transmitting messages were analyzed with different quality of service levels and different payload sizes. The results of their experiment showed that end-to-end delay is related to the message loss with different sizes of payloads.

Moreover, Aazam et al., in a recent survey, evaluated the performance of fog computing using performance metrics such as processing delay, processing costs, and processing power, and derived the performance gains obtained in comparison to a cloud computing-only approach [38]. In the healthcare sector, Alsubaei et al. evaluated security in the Internet of Medical Things (IoMT) [39]. In addition, Kafhali et al. evaluated the response times for accessing medical data stored in a fog-based IoMT implementation framework [40]. They also proposed a queuing model to predict the minimum number of computing resources (both fog and cloud nodes) required to meet the service level agreement (SLA) for response time. Another study in the healthcare field was presented by Vilela et al., who compared the performance of fog-based computing to the conventional cloud computing model in a real-time healthcare monitoring system [41]. Edge Bench is another benchmark for serverless edge computing platforms that is used to measure the performance of two edge computing platforms, Greengrass and Azure [42]. In addition, DeFog, a fog computing benchmark, was proposed to provide a standard methodology and facilitate the understanding of the target platform by collecting a catalogue of relevant metrics for a set of benchmarks [43]; however, most experiments in these studies were carried out using simulators that rely on generic metrics or that focus on one domain, which do not represent real IoT–fog–cloud systems across different domains.

In this study, we extend the previous studies by implementing real-life experiments and analyzing performance metrics from a popular cloud provider (AWS) and IoT protocol (MQTT). We implement two real-life cloud-based IoT environments to measure their performance. In addition, we use different numbers of IoT devices to increase the numbers of subscribers and publishers in order to understand how these factors impact the results.

## 4. Experiment Setup

This section presents the hardware and software components used to set up our experiment. In this paper, we present several IoT–cloud implementation frameworks, namely (1) IoT–cloud, (2) IoT–fog–cloud using a bridge, and (3) IoT–fog–cloud using Python. The hardware and software configurations used in these implementation frameworks are discussed in the following subsections.

### 4.1. Hardware

**Figure 2:**
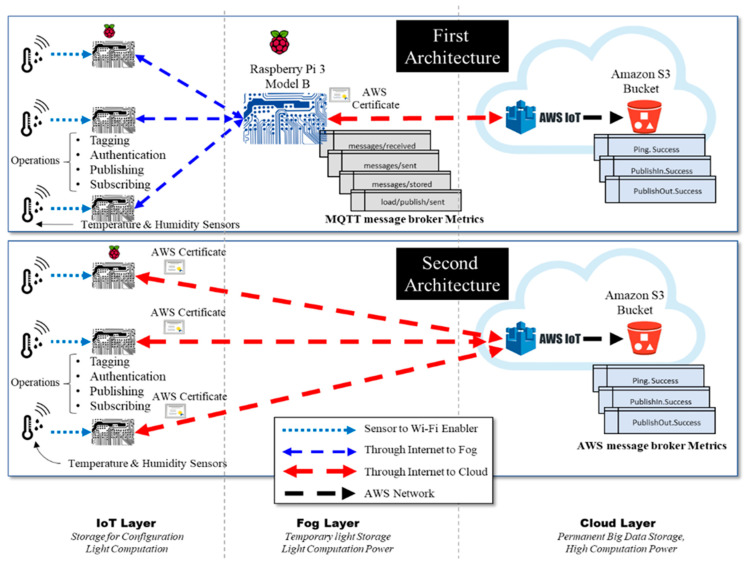
First architecture (IoT–fog–cloud) vs. second architecture (IoT–cloud).

As shown in Figure 2, the experiment discussed in this section involved two architectures. The first architecture is IoT–fog–cloud, while the second is IoT–cloud. In this section, we first describe the devices that were used in the two cloud-based IoT environments, then explain the two architectures in detail. In this experiment, we used DHT11 devices [44] and a Raspberry 3 Pi model B [45]. The DHT11 device is a low-cost sensor that is used to measure the temperature and humidity of the surrounding air. The purpose of the DHT11 sensor in this experiment was to generate real data for the experiment. The Raspberry Pi is a low-cost, single-board computer with built in WiFi and processing capability that is used across several domains, such as in weather monitoring, smart homes, and smart health care. In this experiment, the purpose of the Raspberry Pi was to provide light computation capability to the DHT11 sensor data. In addition, it provided light storage for the DHT11 configurations. Moreover, the Raspberry Pi can be easily moved to different locations. A complete list of the hardware used in this experiment is available in Table 1.

#### 4.1.1. First Architecture

**Figure 3:**
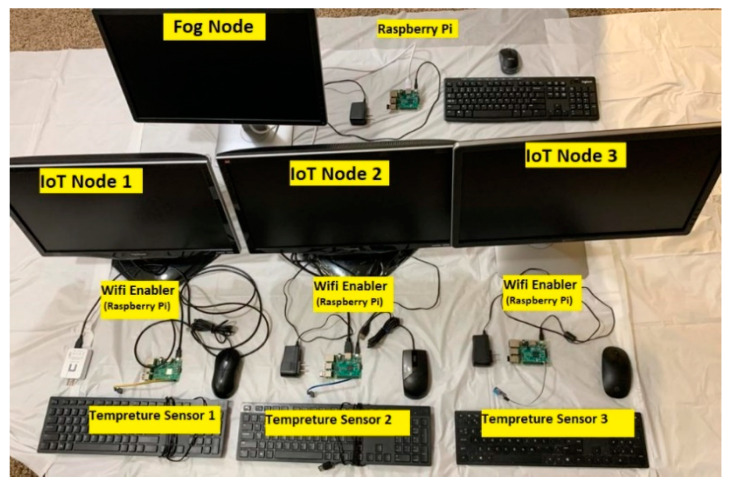
Hardware used in the first architecture (IoT–fog–cloud).

In the first architecture, each DHT11 sensor is connected to only one Raspberry Pi. The Raspberry Pi is used here to enable WiFi connectivity, since the DHT11 sensors are not equipped with network interfaces. Each sensor is connected to Raspberry Pi board (i.e., connectivity enabler), which is considered an IoT device in the IoT layer. Each IoT device is connected via WiFi to another Raspberry Pi board that acts as a fog node in the fog layer. Communication between the IoT devices, the fog node, and the cloud uses the MQTT protocol. For this, an MQTT broker called Eclipse Mosquitto [25] is installed in the Raspberry Pi, acting as a fog device. The Mosquitto MQTT broker exchanges all messages using the subscribe–publish model presented in [1]. The Mosquitto MQTT broker is also used to filter all messages based on topics. A topic refers to an UTF-8 string that the broker (i.e., Mosquitto) uses to filter messages for each connected IoT device. Each data type (i.e., humidity, temperature) in our experiment is considered a separate topic. The data generated by the sensors and collected by the three IoT devices are transmitted over the Internet to the Raspberry Pi acting as the fog node. This Raspberry Pi, which contains the MQTT broker Mosquitto on it, is then connected over the Internet to the AWS cloud. The communication between the three layers occurs through the Internet. Figure 3 shows the hardware used to implement the first architecture.

#### 4.1.2. Second Architecture

In the second architecture, all three IoT devices, consisting of sensors and Raspberry Pi boards (i.e., connectivity enablers), are directly connected to the AWS cloud; therefore, the real data received by device one, device two, or device three are forwarded wirelessly to the AWS cloud layer. The hardware used in the second architecture is identical to that used in Figure 3, except that no fog node is used in this architecture.

### 4.2. Software

We installed Arduino IDE [46] software on top of the three Raspberry Pi boards used to connect the IoT devices. Arduino IDE is a cross-platform application that is written in functions from C and C++ and is used to write and upload programs to Arduino-compatible boards such as Raspberry Pi. We used Arduino IDE to read the data collected by the sensors (i.e., temperature and humidity) and then publish it over the Internet to the fog node. We used C++ scripts in Arduino IDE to perform certain operations on the IoT devices, namely tagging, authentication, publishing, and subscription.

#### 4.2.1. First Architecture

In the first architecture, Algorithm 1 gathers data from the IoT devices and forwards it to the fog. This involves authenticating the IoT devices to communicate with the MQTT broker on the fog device and publish data to it. The following two sections present two ways for fog nodes to communicate with the cloud.
**Algorithm 1.** Gather data generated from the IoT device and forward it to the fog node. First architecture/*. This algorithm authenticates the IoT device to the fog device, generates temperature and humidity data via DHT11 sensors, and publishes them to the Mosquitto MQTT broker on the fog device1: **Define** the Type of DHT Sensor, which is DHT11 2: **Define** the Input/Output Pins of the Raspberry Pi to which the DHT11 is Connected 3: **Define** an Object of the Sensor with Two Arguments: DHT Pin and DHT Type 4: **Define** the Name of the Network 5: **Define** the Password of the Network 6: **Define** the Variable of MQTT Broker 7: **Define** only Two Variables of Humidity Topic and Temperature Topic for each IoT Device (DHT11 + Raspberry Pi) in Each Experiment 8: **Create** Two Instances of Clients, One used to Connect to the Internet and the other used to Connect to the MQTT Broker 9: **Run** MQTT Connection, Setup, Loop 10: **Function:** MQTT Connection 11:    **Connect** to Internet 12:    **If** (the Connection is Established) then 13:      **Print** “Connected” 14:      **Connect** to MQTT Broker on Fog Device 15:    **Else** (the Connection isn’t Established) then 16:        **Try** Reconnecting to Internet 17: **Function:** Setup 18:    **Start** a Serial Communication at 9600 Board Rates 19:    **Initialize** the DHT11 Sensor 20: **While** (MQTT Connection is True): 21:    **Read** Humidity 22:    **Read** Temperature 23:    **Print** Humidity 24:    **Print** Temperature 25:    **Publish** Humidity Topic with Its Read Value to MQTT Broker on Fog Device 26:    **Publish** Temperature Topic with Its Read Value to MQTT Broker on Fog Device 27: **End while**

- Bridging

In our fog-aided IoT implementation, the MQTT broker (i.e., Mosquitto) is installed on top of a Raspberry Pi board that serves as a fog node. In such cases, the MQTT broker needs to be very close to where the sensors are deployed. The Mosquitto MQTT broker has a built-in capability that allows the received data to be sent directly to the cloud (AWS IoT Core) by specifying the address of the AWS IoT core service used. This operation is called bridging (please see [47] for more information). Algorithm 2 illustrates the connection of IoT devices to the AWS IoT core using a bridge connection.
**Algorithm 2.** Bridge every message received from the IoT devices based on the topics of the messages to the AWS broker on the cloud/*. This algorithm authenticates the IoT device to the MQTT broker on the fog device, bridges to the data received from the sensors via the MQTT broker, filters them based on topics, authenticates to the AWS via certificates, and then publishes the filtered data to the AWS IoT core service1: **Define** the Variable of the Endpoint of Amazon Web Service with Port Number 8883 2: **Determine** which Topics of the Messages to Bridge to AWS 3: **Define** the Version of the Protocol to be used between the MQTT Broker and the AWS Broker 4: **Create** One Instance of the Client to be used Over the MQTT Protocol 5: **Define** the Name of the Bridge Connection 6: **Start** Connection 7: **Configure** the Bridge using SSL/TLS Support 8: **Define** Bridge_Cafile to Hold the Path of Amazon Root CA Certificate 9: **Define** Bridge_Certfile to Hold the Path of Amazon Certificate 10: **Define** Bridge_Keyfile to Hold the Path of Private Key

- Python Script

Using the same Mosquitto MQTT broker, we developed a Python script on the fog device to replace the built-in Mosquitto bridging capability to simultaneously authenticate the MQTT broker and AWS, receive the data from the sensors, and filter and publish the data received to the AWS IoT core service. This Python script provides more flexibility for future improvements in security and performance. The following algorithm depicts the operations implemented in our Python script. Algorithm 3 is used to authenticate IoT devices to fog devices, gather the temperature and humidity data from the DHT11 sensors, and publish the data to the Mosquitto MQTT broker on the fog device. The algorithm also authenticates the fog node to interact with AWS IoT Core (i.e., the cloud). Afterward, the algorithm filters the collected data and publishes it to the cloud.
**Algorithm 3.** Receive data from the IoT devices and forward it to the cloud/*. This algorithm authenticates the IoT device to the MQTT broker on the fog device, subscribes to the data received from the sensors via the MQTT broker, filters the data based on topics, authenticates to the AWS via certificates, and then publishes the filtered data to the AWS IoT core service1: **Define** Two Variables (Humidity Topic and Temperature Topic) for each IoT Device in Each Experiment 2: **Define** the Variables of MQTT Broker and MQTT Port 3: **Create** Two Instances of Clients, one used for the MQTT Broker and the other used for the AWS Broker 4: **Connect** the First Client to the MQTT Broker using the IP Address of the Fog Device and MQTT Port 5: **Create a Loop_Start ()** Method to Start a New Thread for the First Client 6: **Set** the Transport Layer Security (TLS) for the Second Client using the Three Paths of AWS Certificates and the Current Version of MQTT Protocol 7: **Connect** the Second client to AWS Broker using AWS Endpoint and AWS Port 8: **Create a Loop_Start ()** Method to Start a New Thread for the Second Client 9: **While** True **do** 10:    **Define** a Connection Function 11:        **Subscribe** for All Topics in each IoT Device 12:        **Print** “Connected” when the Connection is Established 13:        **Print** “Error” when the Connection is Disconnected 14:    **Define** a Message Function 15:      **Get** Topic of The Message 16:      **Get** Payload of the Message 17:      **Print** Topic of the Message 18:      **Print** Payload of the Message 19:      **Publish** Topic and Payload of the Message to AWS Cloud 20:    **Make** the First Client Execute the Two Functions: (1) Connection, and (2) Message 21:    **Make** the Second Client Execute the Message Function to Publish the Recovered Data to AWS Cloud 22: **End while**

#### 4.2.2. Second Architecture

In the second architecture, Arduino IDE is installed on top of the three Raspberry Pi boards used to connect the IoT devices. Arduino IDE is used to read the data collected by the sensors (i.e., temperature and humidity) and then publish it over the Internet directly to the cloud. This operation requires authentication to the cloud before the data can be published. The process used to authenticate the IoT device (i.e., DHT11 + Raspberry Pi) to access the cloud (AWS IoT Core) using the AWS certificate and then to publish the data generated by the sensors to the cloud is illustrated in Algorithm 4.
**Algorithm 4.** Gather data generated from the IoT device and forward it to the cloud—Second architecture/*. This algorithm authenticates the IoT device to the AWS cloud via certificates, generates temperature and humidity data via the DHT11 sensors, and publishes them to the AWS cloud1: **Define** the Type of DHT Sensor, which is DHT11 2: **Define** the Input/Output Pins of the Raspberry Pi to which the DHT11 is Connected 3: **Define** an Object of the Sensor with Two Arguments, DHT Pin and DHT Type 4: **Define** the Name of the Network 5: **Define** the Password of the Network 6: **Define** the Variable of Endpoint of Amazon Web Service 7: **Define** only Two Variables (Humidity Topic and Temperature Topic) for each IoT Device (DHT11 + Raspberry Pi) in Each Experiment 8: **Create** a client to Connect to AWS using AWS Endpoint and Port Number 8883 9: **Run** Connection, Setup, Loop 10: **Function:** Connection 11:    **Connect** to Internet 12:    **If** (the Connection is Established) **then** 13:      **Print** “Connected” 14:    **Else** (the Connection isn’t Established) **then** 15:      **Try** Reconnecting to Internet 16: **Function:** Setup 17:    **Start** a Serial Communication at 9600 Board Rates 18:    **Initialize** the DHT11 Sensor 19:    **Run** a Connection Function 20:    **Convert** the AWS Certificates to der Format 21:    **Open** Certificate 22:    **If** (Certificate is Existing) 23:      **Load** Certificate 24:    **Else** 25:      **Print** “Certificate is not Existing” 26:    **Open** Amazon Root CA Certificate 27:    **If** (Amazon Root CA Certificate is Existing) 28:      **Load** Amazon Root CA Certificate 29:    **Else** 30:      **Print** “Amazon Root CA Certificate is not Existing” 31:    **Open** Private Key 32:    **If** (Private Key is Existing) 33:      **Load** Private Key 34:    **Else** 35:      **Print** “Private Key is Not Existing” 36: **While** (Connection is True): 37:    **Read** Humidity 38:    **Read** Temperature 39:    **Print** Humidity 40:    **Print** Temperature 41:    **Publish** Humidity Topic with its Read Value to MQTT Broker on Fog Device 42:    **Publish** Temperature Topic with its Read Value to MQTT Broker on Fog Device 43:    **End while**

To monitor the Mosquitto MQTT broker, Algorithm 5 illustrates how $SYS-Topics is used to provide metrics.
**Algorithm 5.** Monitoring script on fog device/*. This algorithm connects to the MQTT broker, subscribes to SYS-Topics via the MQTT broker to monitor and provide benchmark metrics of the Mosquitto broker, and publishes the results based on these metrics1: **Create** One Instance of Clients to Connect to Mosquitto Broker 2: **Connect** the Client to MQTT Broker using the IP Address of Fog Device and MQTT Port 3: **Define** a Connection Function 4:    **Print** “Connected” when the Connection is Established 5:    **Print** “Error” when the Connection is Disconnected 6:    **Subscribe** To $SYS/# Topics to Monitor the Mosquito MQTT Broker (Print the Metrics Results Of Mosquitto Broker On Fog Device 7: **Make** the Client Execute the Connection Function 8: **Create a Loop Forever ()** Method for the Client to Remain Monitoring the Mosquitto

## 5. Descriptions of Metrics

Many metrics can be used to measure the performance in IoT systems. The following subsections describe the metrics that we utilized to measure the performance in the cloud and fog layers.

### 5.1. Cloud Layer: AWS IoT Metrics

Many metrics can be used to measure the performance of cloud-based IoT systems from the cloud layer. Since we used the AWS IoT as the cloud service provider for our experiments, we used Amazon CloudWatch to measure the performance. Amazon CloudWatch has been used in the literature to monitor performance [48,49,50]. Amazon CloudWatch processes and analyzes data in real time and provides the following metrics to measure our two cloud-based IoT environments:

- Connect Success: This metric is used to collect the number of successful connections from our IoT nodes or fog nodes to the AWS message broker.
- Ping Success: This metric is used to collect the number of ping messages received by the AWS message broker. These ping messages are received from the fog node(s) in the first architecture and from the IoT node(s) in the second architecture.
- PublishIn Success: This metric is used to collect the number of publish requests successfully processed by the AWS message broker. As with the ping messages, these messages are received from the fog node(s) in the first architecture and from the IoT node(s) in the second architecture.
- PublishOut Success: This metric is used to collect the number of publish requests successfully made by the AWS message broker to the fog nodes in the first architecture and to the IoT nodes in the second architecture.
- Subscribe Success: This metric is used to collect the number of successful subscribe requests processed by the AWS message broker. These requests are made by the fog node in the first architecture and made directly by the IoT devices in the second architecture.
- PublishIn Clienterror: This metric is used to collect the number of publish requests rejected because they did not meet the AWS IoT requirements.
- Unsubscribe Success: This metric is used to collect the number of unsubscribe requests that were successfully processed by the AWS message broker. These unsubscribe requests are made by the fog node in the first architecture and made directly by the IoT devices in the second architecture.
- Throttle Exceeded: This metric is used to collect the number of requests that have been throttled because the client (i.e., the IoT node or fog node) has sent too many messages and exceeded the allowed message rate.
- PublishOut Throttle: This metric is used to collect the number of publish requests that have been throttled because the client (i.e., IoT node or fog node) has exceeded the allowed message rate.

### 5.2. Fog Layer: Eclipse Mosquitto Broker Metrics

Many metrics can be used to measure the performance of the fog layer. MQTT has been used in the literature as a lightweight protocol to communicate between messages [34,35,51]. Since we used the MQTT protocol for the message broker on the fog node, we utilized Eclipse Mosquitto [25] in our experiments. Eclipse Mosquitto [25] provides some metrics as $SYS topics, which are described below:

- $SYS/broker/uptime: This metric is used to measure the amount of time in seconds the broker has been online.
- $SYS/broker/load/messages/received: This metric is used to measure the moving average of the number of all types of MQTT messages received by the broker over different time intervals.
- $SYS/broker/load/messages/sent: This metric is used to measure the moving average of the number of all types of MQTT messages sent by the broker over different time intervals.
- $SYS/broker/load/publish/received: This metric is used to measure the moving average of the number of publish messages received by the broker over different time intervals.
- $SYS/broker/load/publish/sent: This metric is used to measure the moving average of the number of publish messages sent by the broker over different time intervals.
- $SYS/broker/load/bytes/received: This metric is used to measure the moving average of the number of bytes received by the broker over different time intervals.
- $SYS/broker/load/bytes/sent: This metric is used to measure the moving average of the number of bytes sent by the broker over different time intervals.
- $SYS/broker/load/sockets: This metric is used to measure the moving average of the number of socket connections opened to the broker over different time intervals.
- $SYS/broker/load/connections: This metric is used to measure the moving average of the number of CONNECT packets received by the broker over different time intervals.
- $SYS/broker/messages/stored: This metric is used to measure the number of messages currently held in the message store. This includes retained messages and messages queued for durable clients.
- $SYS/broker/store/messages/bytes: This metric is used to measure the number of bytes currently held by message payloads in the message store. This includes retained messages and messages queued for durable clients.
- $SYS/broker/subscriptions/count: This metric is used to measure the total number of subscriptions active on the broker.
- $SYS/broker/heap/current: This metric is used to measure the current size of the heap memory in use by Mosquitto.
- $SYS/broker/messages/received: This metric is used to measure the total number of messages of any type received since the broker started.
- $SYS/broker/messages/sent: This metric is used to measure the total number of messages of any type sent since the broker started.
- $SYS/broker/publish/messages/received: This metric is used to measure the total number of PUBLISH messages received since the broker started.
- $SYS/broker/publish/messages/sent: This metric is used to measure the total number of PUBLISH messages sent since the broker started.
- $SYS/broker/bytes/received: This metric is used to measure the total number of bytes received since the broker started.
- $SYS/broker/bytes/sent: This metric is used to measure the total number of bytes sent since the broker started.
- $SYS/broker/publish/bytes/received: This metric is used to measure the total number of PUBLISH bytes received since the broker started.
- $SYS/broker/publish/bytes/sent: This metric is used to measure the total number of PUBLISH bytes sent since the broker started.

## 6. Analysis Methods

We used two benchmark metrics to analyze the performance of the two IoT architectures implemented in this paper. In the two cloud-based IoT architectures, we set the number of subscribers and publishers to two for each device. This is because the IoT devices (i.e., the DHT11 sensors) generate two types of data, namely (1) temperature and (2) humidity data; therefore, as the number of sensor devices increase, the number of subscribers and publishers should also increase. This provides more accurate, consistent, and real results about the environment performance and scalability. In this section, we present the methods that we used to perform the experiments on both architectures.

### 6.1. Architecture 1 vs. Architecture 2

The performance of the two cloud-based IoT architectures was analyzed using the AWS benchmark and the obtained results were compared. The experiment was performed using a different number of IoT devices each time (1, 2, or 3 IoT devices) to compare and analyze the results in order to show the impacts of the fog layer in the first architecture on IoT environments. Figure 2 shows the experimental setup and metrics applied to measure the performance of the two cloud-based IoT environments. AWS, as with any IoT platform provider, requires that any device be authenticated before communicating with it. AWS uses certificates to authenticate devices. As shown in Figure 2, the location where the certificate is stored is different in the two architectures due to the structure of the environments. Since the first architecture has a fog layer between AWS and the IoT devices, the certificate is stored in the fog device (i.e., the Raspberry Pi board serving as the fog layer). In the second architecture, however, since the IoT devices are directly connected to AWS, the certificates are stored in each IoT device.

### 6.2. Architecture 1 Implementation: Python Script vs. Bridging

**Figure 4:**
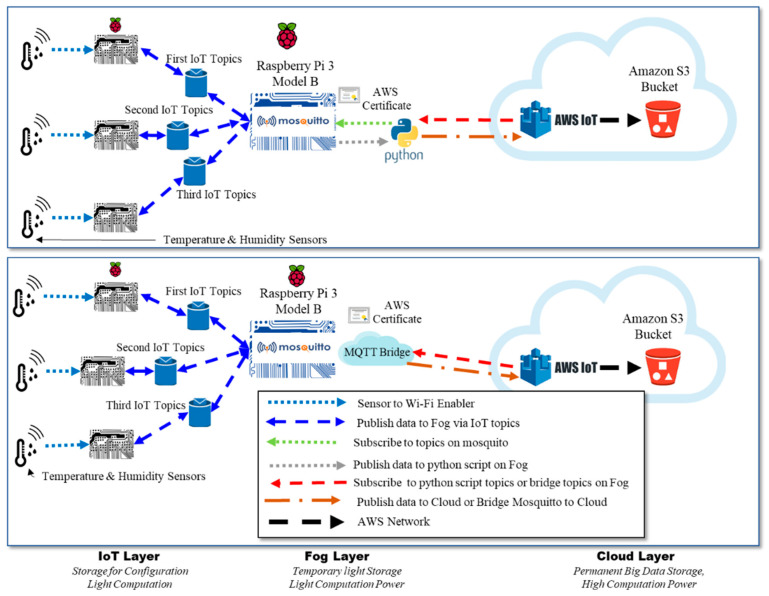
IoT–fog–cloud architecture using two methods: Python script and MQTT bridging.

The first architecture of the IoT–fog–cloud layer was implemented using two different experiments, as shown in Figure 4. The first experiment was based on a Python script shown in Algorithm 3 that manually receives, filters, and forwards the messages to the cloud. The second experiment (Algorithm 2) bridges all of the messages received from the IoT devices based on their topics to AWS. The purpose of using the bridge in the first architecture is to connect two brokers, the MQTT message broker “mosquitto” and the AWS message broker, to exchange messages based on the different topics and to validate the results of the first architecture. AWS benchmarks were used to analyze the performance of the first architecture in two different implementation frameworks. Although bridging allows faster implementation, the Python script provides more flexibility to add features to optimize performance. The objective of this analysis is to show the impacts of both implementation frameworks.

### 6.3. Architecture 1 Measurement: Mosquitto Metrics vs. AWS Metrics

**Figure 5:**
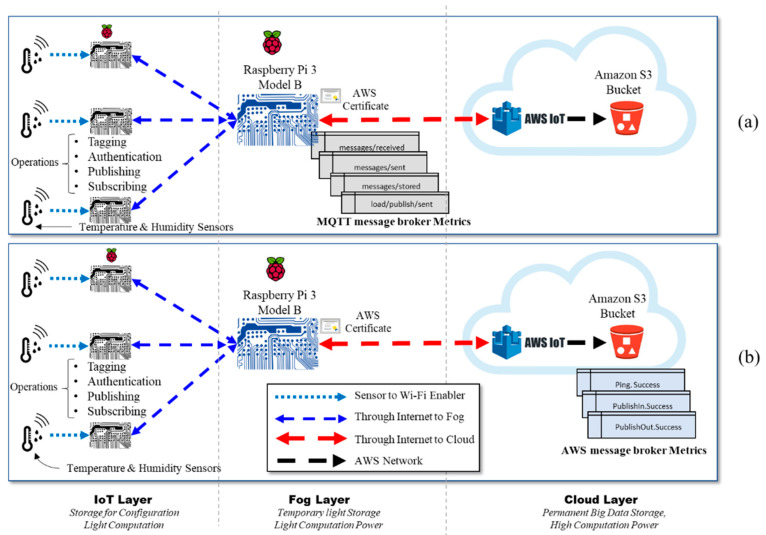
(**a**) Metrics applied in the fog layer vs. (**b**) metrics applied in the cloud layer.

The performance of the first architecture (i.e., IoT–fog–cloud) was measured from both the fog side and the cloud side. The fog-side metrics (i.e., Mosquitto broker metrics) were measured over different durations (30 s, 1 min, 5 min, 15 min, 30 min, 45 min, and 1 h), while the cloud-side metrics (i.e., AWS metrics) were measured over 30 s, 1 min, 5 min, 15 min, and 1 h (due to the AWS platform constraints, it was difficult to unify the experiment durations). Figure 5 presents the different metrics used to measure the performance of the IoT environment that uses a fog layer from two sides. Figure 5a presents the metrics applied to measure the performance of the IoT environment at the fog layer, whereas Figure 5b presents the metrics applied to measure the performance of the IoT environment at the cloud layer. This was done to ensure that the performance of the IoT environment remains consistent in the fog and cloud layers using different setups.

## 7. Results and Description of Experiments

In this section, the results of the two architectures of cloud-based IoT environments will be analyzed using the two benchmarks metrics (Mosquitto and AWS) based on the selected architecture using one, two, or three IoT devices with increasing numbers of subscribing and publishing requests. In the following sections, the benchmark metrics of each architecture are described in detail, along with observations.

### 7.1. Descriptions of the Three Experiments on the First Architecture with One, Two, or Three IoT Devices Using AWS Benchmark Metrics (Cloud Layer)

The Amazon CloudWatch monitor has a variety of metrics that can be used to analyze the cloud layer, which were used in the experiments on the first architecture, as shown in Table 2 and Table 3. The AWS metrics are (1) Connect Success, (2) Ping Success, (3) PublishIn Success, (4) PublishOut Success, (5) Subscribe Success, (6) Unsubscribe Success, (7) PublishIn Clienterror, (8) Throttle Exceeded, and (9) PublishOut Throttle. These metrics are explained in detail in Section 5.1. The metrics were collected for one hour, starting from 30 s, as shown in Table 2 and Table 3. Before analyzing the results obtained using the AWS metrics, a number of assumptions should be taken into consideration. First, the number of successful connections from either IoT devices or fog devices using the Connect Success metric must be equal to the number of subscribe requests received from either fog devices or IoT devices using the Subscribe Success metric because the loss of connections will lead to the loss of subscribe requests in each device and the subscriptions will be renewed automatically once the connection is reestablished. Second, the number of unsubscribe requests received from either IoT devices or fog devices using the Unsubscribe Success metric must reflect the numbers of subscription and publishing requests generated in each IoT device. In our experiments, there were three IoT devices, each of which generated two types of data, namely temperature and humidity. Asusch, the number of IoT devices was three and the number of all subscription and publishing requests was six. Third, the number of publishing requests received from either fog devices or IoT devices using the Publishin Success metric should be close to or the same as the number of publish requests made by AWS to either fog devices or IoT devices using the Publishout Success metric.

The experiments using the first architecture, which included a fog layer, were conducted with one, two, or three IoT devices for different time periods (30 s, 1 min, 5 min, 15 min, and 1 h), as shown in Table 2. The first experiment involved one IoT device attached to one fog device, with the results showing that the Connect Success and Subscribe Success numbers reflected the numbers of subscribing and publishing requests for one IoT device, with two subscribe and publish requests. This was because the connection was not disconnected and the subscribe request was not lost from either the IoT or the fog device. Moreover, there was only one IoT device connected to only one fog device, which was then authenticated to the AWS cloud, and no other devices interrupted them. In addition, the PublishIn Success and PublishOut Success numbers were expected and stable because the data came from the IoT device and were filtered based on the fog device, then sent to the AWS cloud. It takes time for the data to be transferred between the three layers of IoT, fog, and cloud. In the second experiment, on the other hand, two IoT devices were attached to the fog device, as shown in Table 2. The results indicated that the Connect Success and Subscribe Success numbers were equal to the numbers of subscribing and publishing requests for both IoT devices, since two IoT devices were linked to one fog device with four subscribing and publishing requests; therefore, the PublishIn Success and PublishOut Success numbers were significantly increased with two IoT devices compared to the previous experiment using only one IoT device attached to one fog device. This was because two IoT devices were connected to only one fog device serving as the middle layer between the IoT devices and the AWS cloud, which was used to authenticate to the AWS cloud; thus, the fog device increased the number of messages published to the AWS cloud and reduced the number of IoT devices that needed to be authenticated to the AWS cloud. The third experiment used three IoT devices connected to one fog device, as shown in Table 2. The Connect Success and Subscribe Success numbers still reflected the actual numbers of subscribing and publishing requests for all three IoT devices, even though there was only one fog device; thus, the PublishIn Success and PublishOut Success numbers were significantly increased due to the high volumes of published messages from the three IoT devices. As such, the fog device made a significant impact in filtering and transferring the actual volumes of data from the three IoT devices to the AWS cloud and decreasing the number of authentications required for the AWS cloud.

### 7.2. Descriptions of the Three Experiments on the Second Architecture with One, Two, or Three IoT Devices Using AWS Benchmark Metrics (Cloud Layer)

In Table 2, it can be seen that for one IoT device, the Connect Success and Subscribe Success numbers are low because there is only one IoT device with two subscribe and publish requests authenticated to the AWS and with no other IoT devices attached with it; thus, the connection and subscription are established at the same time. In addition, the PublishIn Success and PublishOut Success numbers are consistent and high due to the single authenticated IoT device. With two IoT devices with four subscribe and publish requests, however, the Connect Success and Subscribe Success numbers increase because there are two IoT devices attempting to authenticate to the AWS cloud at one time, which increases the number of connections and subscriptions. The PublishIn Success and PublishOut Success numbers are not substantially change compared to the previous experiment even though the numbers of subscribe and publish requests increase when the second IoT device is added because the second IoT device tries to authenticate to the AWS cloud while the first IoT device tries to publish messages to the AWS cloud. As a result, publish messages are lost in each period. The third experiment, using three IoT devices with six subscribe and publish requests, shows that the number of connections and subscriptions increases while the PublishIn Success and PublishOut Success numbers are low. This is because the three IoT devices try to authenticate to the AWS cloud at once, causing a huge number of connection and subscription requests.

### 7.3. Descriptions of the Three Experiments on the First Architecture with One, Two, or Three IoT Devices Using Mosquitto Benchmark Metrics (Fog Layer)

The MQTT Mosquitto broker using SYS-Topics has several metrics that were used in the experiments on the first architecture from the fog layer, as shown in Table 4, Table 5 and Table 6. The Mosquitto broker metrics are (1) load/messages/received, (2) load/messages/sent, (3) load/publish/received, (4) load/publish/sent, (5) load/bytes/received, (6) load/bytes/sent, (7) load/sockets, (8) load/connections, (9) messages/stored, (10) store/messages/bytes, (11) subscriptions/count, (12) heap/current, (13) messages/received, (14) messages/sent, (15) publish/messages/received, (16) publish/messages/sent, (17) bytes/received, (18) bytes/sent, (19) publish/bytes/received, and (20) publish/bytes/sent. These metrics are described in detail in Section 5.2 and were obtained over one hour, starting from 30 s, as shown in Table 4, Table 5 and Table 6. Before analyzing the Mosquitto broker metrics on the fog device, a number of hypotheses should be discussed. First, the number of subscribe requests received from each IoT device using the subscription/count metric must reflect the number of subscribe and publish requests established in each IoT device. Second, the number of publish messages received from each IoT device in the first architecture using the publish/messages/received metric should be equal to the number of publish messages received from the fog device using the Publishin Success metric. Third, the number of publish messages sent by the Mosquitto broker on the fog device using the publish/messages/sent metric should increase as the number of IoT devices increases due to the broker capabilities and the high computational power of the fog device to process and publish many messages. The experiments on the first architecture, which had a fog device between the IoT device(s) and the AWS cloud, were performed using one, two, or three IoT devices for different periods of 30 s, 1 min, 5 min, 15 min, 30 min, 45 min, and 1 h. The first experiment involved one IoT device attached to the fog device connected to the AWS cloud, as shown in Table 4. The results showed that the number of subscribe requests using the subscriptions/count metric on the fog device reflected the number of subscribe and publish requests of the single IoT device with two subscribe and publish requests. This was because the fog device subscribes to the topics of each message received from IoT devices, filters them based on the topics of the messages, then publishes them to the AWS cloud. As such, each message is identified by its topic in each layer. In addition, the number of publish messages made by the fog device (publish/messages/sent) is high, even though there is only one IoT device, with two subscribe and publish requests. This is because the fog device has high capability to filter and publish many messages, since it is located close to the IoT device that is generating the real data. Moreover, the size of the heap memory used by Mosquitto on the fog device is stable over different durations from 30 s to 1 h. The second experiment was conducted using two IoT devices with four subscribe and publish requests attached to the fog device, which connects to the AWS cloud, as shown in Table 5. The results showed that the number of subscribe requests increased since the number of IoT devices increased to two. In addition, the number of publish messages made by the fog device (publish/messages/sent) significantly increased because two IoT devices with four subscribe and publish requested were attached. The third experiment was conducted using three IoT devices with six subscribe and publish requests attached to a fog device and then to the AWS cloud, as shown in Table 6. The results demonstrated that the number of subscribe requests was six when the number of IoT devices increased to three. In addition, the number of publish messages made by the fog device (publish/messages/sent) was significantly higher when the number of IoT devices increased to three.

## 8. Evaluation of Results

### 8.1. First Architecture vs. Second Architecture

**Figure 6:**
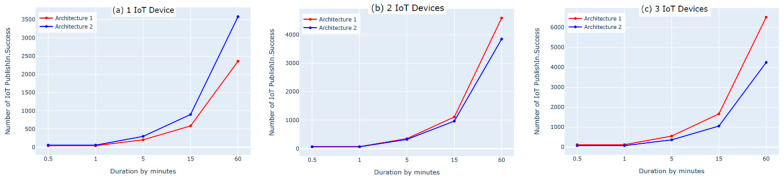
AWS IoT message broker PublishIn Success metrics with 1, 2, and 3 IoT devices on the North Virginia datacenter (cloud layer).

**Figure 7:**
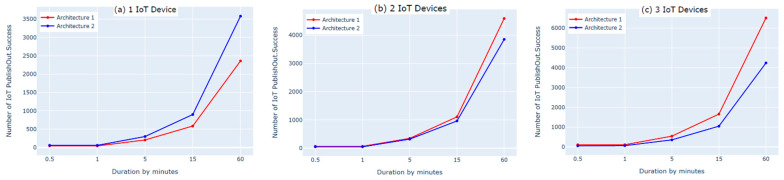
AWS IoT message broker PublishOut Success metrics with 1, 2, and 3 IoT devices on the North Virginia datacenter (cloud layer).

**Figure 8:**
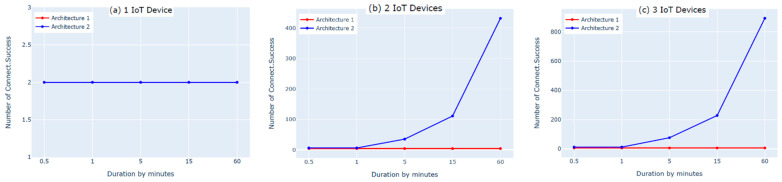
AWS IoT message broker Connect Success metrics with 1, 2, and 3 IoT devices on the North Virginia datacenter (cloud layer).

**Figure 9:**
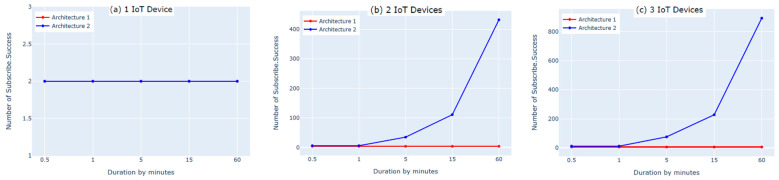
AWS IoT message broker Subscribe Success metrics with 1, 2, and 3 IoT devices on the North Virginia datacenter (cloud layer).

The first experiment on each of the two architectures was performed by connecting one IoT device to the cloud. We used two subscribe and two publish commands to send the data. The results showed that the numbers of subscribe and publish commands were the same for both architectures and matched the defined numbers of subscribe and publish commands for one IoT device; however, the number of published messages (PublishIn Success and PublishOut Success, shown in Figure 6a and Figure 7a) in the first architecture was slightly lower than the number of published messages in the second architecture. This was because there was an additional hop (i.e., fog layer) in the middle of the first architecture that processed the messages before transmitting them to the AWS cloud. As a result, the messages took longer to be delivered to the AWS cloud. In contrast, the second architecture did not have a fog layer and the messages were forwarded directly to the AWS cloud, so the number of published messages was slightly higher. Moreover, the IoT devices must be authenticated to the AWS before publishing the messages. The Connect Success and Subscribe Success metrics shown in Figure 8a and Figure 9a show that when using one IoT device, authentication did not impact these two metrics in either architecture.

The second experiment was conducted using two IoT devices and four subscribe and publish commands. The results show that the number of subscribe and publish commands matches the defined number of subscribe and publish commands for two IoT devices only in the first architecture, as shown in Figure 8b and Figure 9b (Connect Success and Subscribe Success); the number of subscribe and publish commands was significantly increased in the second architecture. This is because in the second architecture, the two IoT devices need to be authenticated separately to the AWS cloud, whereas in the first architecture, only one device (the fog node) needs to be authenticated because the IoT devices are authenticated to the fog node in a different, simpler process. Additionally, this high number of connect and subscribe requests in the second architecture causes a loss in the number of messages published to the AWS cloud. This is because while one of the IoT devices is subscribed and publishing, the other device remains trying to connect, as shown in Figure 6b and Figure 7b (PublishIn Success and PublishOut Success). Notably, the rate of published messages in the first architecture is much better than in the second architecture. This is because in the first architecture, there was no failure to subscribe and the fog node was always able to publish messages successfully to the AWS cloud, as shown in Figure 8b and Figure 9b (Connect Success and Subscribe Success).

In the third experiment, we used three IoT devices with six subscribe and publish commands. The results show that the number of subscribe and publish commands matches the defined number of subscribe and publish commands (PublishIn Success and PublishOut Success in Figure 6c and Figure 7c) in the first architecture. In the second architecture, however, the number of subscribe and publish commands is significantly higher. This is because the fog node in the first architecture authenticates three IoT devices and the AWS cloud authenticates only the fog node. In contrast, in the second architecture, the AWS cloud authenticates three IoT devices separately, which increases the number of subscribe and publish commands. This also negatively affects the number of published messages in the second architecture due to the time spent by the IoT devices that are not connected trying to connect, as shown in Figure 6c and Figure 7c (PublishIn Success and PublishOut Success). In contrast, in the first architecture, there is no loss in the published messages, as there is no sign of failure in the number of subscribe and publish commands, as shown in Figure 8c and Figure 9c (Connect Success and Subscribe Success).

In this experiment, we found that the performance of the second architecture was better than that of the first architecture when using one IoT device. However, when using more than one IoT device, the first architecture outperforms the second architecture in terms of performance. Moreover, when using more than one IoT device, the resource utilization in the first architecture was better than in the second architecture because all of the IoT devices were able to successfully connect to the fog node simultaneously. Overall, with an increased number of IoT devices, the first architecture outperforms the second architecture.

### 8.2. Architecture 1 Implementation: Python Script vs. Bridging

**Figure 10:**
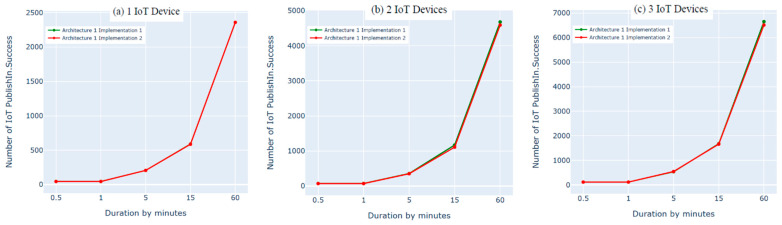
AWS IoT message broker PublishIn.Success metric with 1, 2, and 3 IoT devices on North Virginia datacenter (cloud layer).

**Figure 11:**
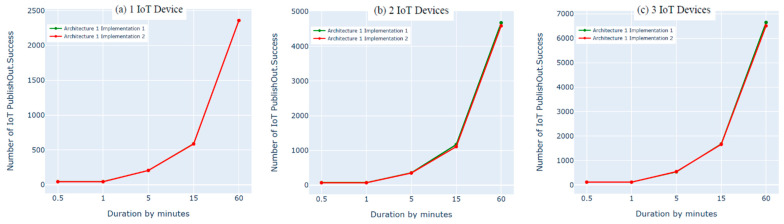
AWS IoT message broker PublishOut.Success metric with 1, 2, and 3 IoT devices on North Virginia datacenter (cloud layer).

**Figure 12:**
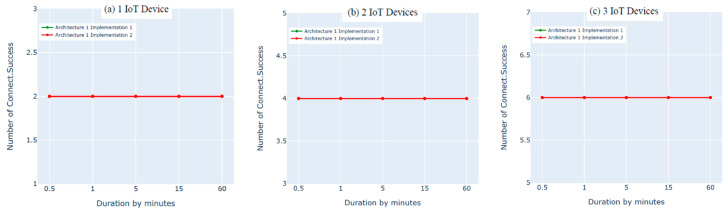
AWS IoT message broker Connect.Success metric with 1, 2, and 3 IoT devices on North Virginia datacenter (cloud layer).

**Figure 13:**
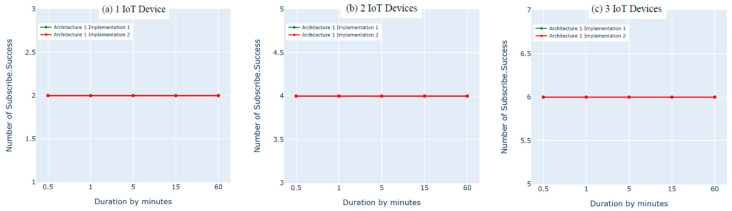
AWS IoT message broker Subscribe.Success metric with 1, 2, and 3 IoT devices on North Virginia datacenter (cloud layer).

In this experiment, we evaluated the first architecture (i.e., IoT–fog–cloud) using two different implementation frameworks (Python Script vs. bridging) using AWS metrics. Three experiments were performed using one, two, or three IoT devices. Using two subscribes and two publishes per device, both implementation frameworks showed insignificant differences in performance using the AWS benchmark, as shown in Figure 10, Figure 11, Figure 12 and Figure 13. Comparing the performance results of the same architecture using two different implementation frameworks shows that the results of our experiment are accurate.

### 8.3. Architecture 1 Measurement: Mosquitto Metrics vs. AWS Metrics

In this section, we evaluate the first architecture of the cloud-based IoT environment using AWS and Mosquitto benchmarks. This is done to prove that we monitored the first architecture from two sides, the cloud layer and the fog layer, as shown in Figure 4.

**Figure 14:**
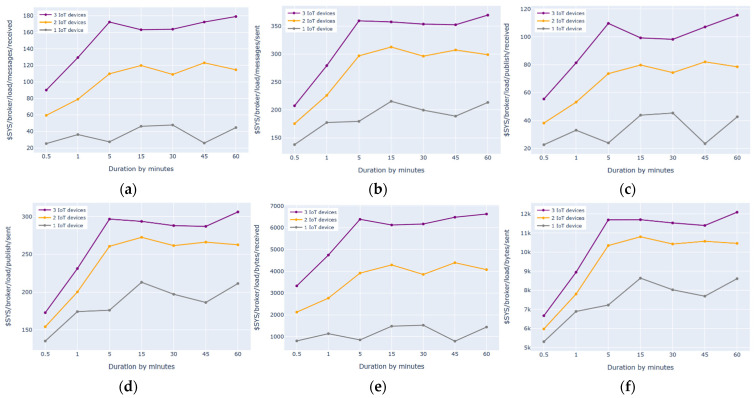

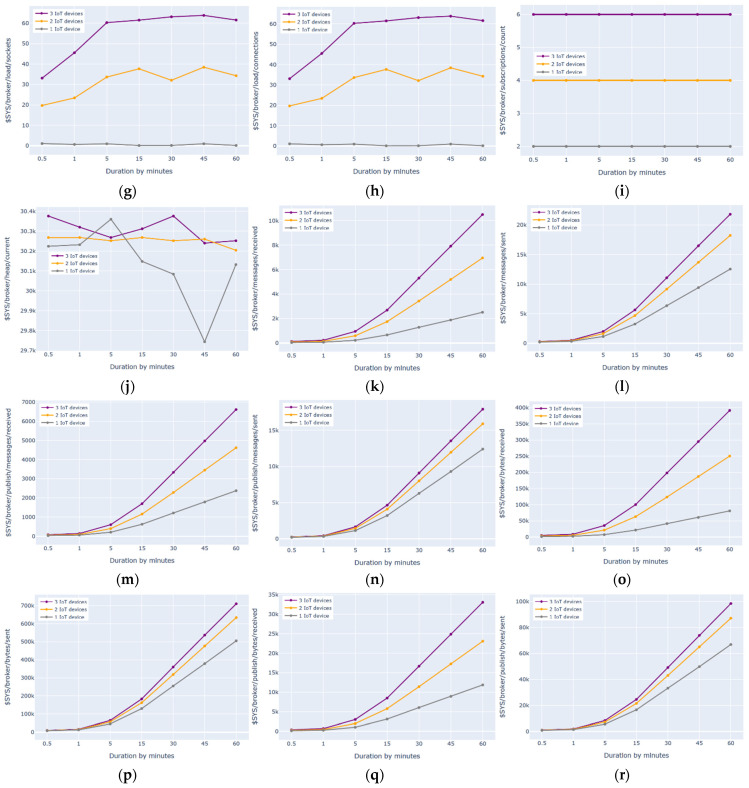
Mosquitto message broker metrics on the fog layer using bridge (Architecture 1).

The first experiment was conducted using one IoT device with two subscribe and two publish commands. The results showed that the number of subscribe and publish commands were the same for both the AWS (i.e., Subscribe Success and Connect Success) and Mosquitto (i.e., $SYS/broker/subscriptions/count) benchmarks and reflected the defined numbers of subscribe and publish commands for one IoT device, as shown in Figure 8a, Figure 9a and Figure 14i. This experiment was implemented using the subscribe–publish mode presented in [1]. In the architecture with three layers (IoT, fog, and cloud), the fog node subscribes to all of the message topics sent from the IoT device(s). These messages are then processed and published to the AWS cloud. Again, the cloud layer (i.e., the AWS cloud) subscribes to all of the message topics sent from the fog device. This is important, as it ensures that the IoT device(s) are connected and able to send data to the fog and then to the cloud without loss.

The number of published messages on the fog device using the Mosquitto metric (publish/messages/received) is equal to the number of published messages on the AWS cloud using the AWS metric (PublishIn Success), as shown in Figure 6a and Figure 14m. This is because the messages generated from one IoT device are received by both the fog and the AWS cloud and no messages are lost during transmission; however, the number of messages processed by the Mosquitto broker ($SYS/broker/publish/messages/sent) on the fog device is significantly higher than the number of messages processed by the AWS broker (PublishOut Success) on the AWS cloud, as shown in Figure 7a and Figure 14n. This is because the fog device is closer to the IoT devices, which reduces the latency of transmitting all of the messages to the AWS cloud. In addition, fog devices play an important role in decreasing the computation overhead caused by the IoT devices due to its close proximity. It also reduces AWS resource consumption by bringing computation closer to the IoT devices; therefore, the memory consumption ($SYS/broker/heap/current) on the fog node remains stable even when the number of processed messages increases, as shown in Figure 14j.

The second and third experiments on the first architecture were performed using two or three IoT devices with four or six subscribe and publish commands, respectively. The results showed that the number of subscribe and publish commands remained identical using the Mosquitto metric ($SYS/broker/subscriptions/count) and the AWS metrics (Subscribe Success and Connect Success) and reflected the defined number of subscribe and publish commands, as shown in Figure 8b,c, Figure 9b,c and Figure 14i. In addition, the number of published messages on the fog device remained equal to the number of published messages on the AWS cloud, as shown in Figure 6b,c and Figure 14m; however, we noticed that when we increased the number of IoT devices, the number of messages sent and processed by the Mosquitto broker on the fog device significantly increased without affecting the performance, as shown in Figure 14n.

Overall, in the first experiment with one IoT device, the number of messages processed by the fog device (i.e., received from the IoT device and sent to the cloud) was higher than the number of messages received by the AWS cloud. Similarly, as the number of IoT devices increased (i.e., using two and three IoT devices), the number of messages made by the Mosquitto broker on the fog device remained higher than the number of messages made by the AWS broker on the AWS cloud. This was because the messages were transmitted through the three layers of the environment (i.e., IoT, fog, and cloud); thus, it took more time for the messages to be delivered to the AWS cloud due to the additional intermediate fog layer. Moreover, since the fog device was located close to the IoT devices, message latency decreased and the processing of the messages on the fog increased. As such, using fog computing was very beneficial when connecting more than one IoT device to the cloud. Overall, as the number of IoT devices increases, the process ability of the fog device in the first architecture outperforms that of the AWS cloud.

## 9. Threats to Validity

The experiments in this paper were implemented using real commercial sensors interacting with a real-world cloud through a commercial IoT service. The environment was vulnerable to real attacks and simulates a real-life IoT environment. We used Raspberry Pi boards to enable the sensors to send collected data through the Internet to the cloud or the fog node. We also used a Raspberry Pi board as a fog node and as the intermediate layer between the sensors and the cloud in the first architecture. The first architecture consisted of three layers (IoT, fog, and cloud), whereas the second architecture consisted of only two layers (IoT and cloud). Every layer operated on a separate network and all networks were connected to the Internet to simulate real-life implementation frameworks. In both architectures, up to three sensors were used to capture temperature and humidity data and send it to the cloud.

Each experiment was performed for an hour, during which the data captured by the sensors were sent to the cloud (i.e., the AWS North Virginia datacenter) and stored there. It would be of interest to investigate whether the results can be applied to other cloud service providers or to other datacenters in different geographical areas. In addition, three sensors were used to conduct the experiments by either connecting the sensors to the cloud directly or by connecting the sensors through a fog node to the cloud. Further experiments are needed to determine how many sensors a fog node can handle before the performance is impacted.

Moreover, our results indicate that the first architecture outperforms the second architecture in publishing the data captured by the sensors to the cloud. This is attributed to the fact that in the second architecture, all of the sensors need a certificate for authentication, whereas in the first architecture, only one certificate for authentication is needed and is placed on the fog node. Notably, this leaves the communication between the IoT layer and the fog layer without a proper authentication mechanism. We plan to utilize the computation and storage capability of the fog node to implement an authentication and authorization model for the sensors that interact with the fog nodes in the future.

## 10. Discussion and Limitations

Fog computing has evolved to support cloud-based IoT environments in many ways. It is known for its ability to lower the communication latency, optimize the communication bandwidth, and enable higher network scalability and heterogeneity [52]. In addition to these advantages, fog computing has many valuable characteristics, such as fog node mobility and location awareness, in addition to the computational ability that IoT devices lack. In this paper, we demonstrated that fog computing has a substantial impact on cloud-based IoT environments in reducing latency and improving communication performance. In addition to these benefits, fog computing has a great deal of advantages that were not extensively discussed in our results.

First, fog-computing-aided IoT environments are known for their higher scalability, since every group of IoT devices is connected to a fog node. This hierarchical structure enables better management, tracking, and monitoring of IoT devices. In addition to this, compared to environments in which IoT devices are connected directly to the cloud, fog-aided IoT environments improve resource utilization. This is attributed to the savings in processing capability, which are wasted when the cloud authenticates a massive number of IoT devices. At first sight, one might say that the same processing time and resources will be consumed at the fog node to authenticate IoT devices. While this is true, with a fog node, consumers of cloud services will not be overcharged for services that are wasted on the cloud to authenticate a significant number of IoT devices, especially because providers charge based on consumption.

From a security standpoint, fog computing spreads risks across distributed fog nodes in fog-aided IoT environments. In addition, authenticating IoT devices at the fog layer provides more flexibility by adding sophistication to the authentication and authorization process, for example through encryption-based access control.

Although the convenience of having a fog layer with semi-heavyweight computation capability has a higher capital cost, in the long run it saves a lot of resources, time, and money.

## 11. Conclusions and Future Works

In this paper, we proposed two architectures of cloud-based IoT environments using a real environment. In one architecture, we used a fog layer between IoT devices and the cloud, whereas in the second, IoT devices published data directly to the cloud. In order to validate our results, we also examined two ways of implementing fog-aided IoT–cloud environments, namely (1) bridging and (2) using a Python script to forward the data to the cloud. For each architecture, we conducted several experiments and increased the number of IoT devices as well as the number of subscribe and publish commands in each experiment. To evaluate the experimental performance, we used two sets of benchmark metrics, namely (1) AWS message broker metrics and (2) Mosquitto message broker metrics. The performance was evaluated based on the following analysis methods. First, we compared the performance of the first and second architectures. Second, we compared the performance of the two implementation frameworks of the fog-aided IoT environments (i.e., Python script vs. bridging). Finally, to validate our results, the performance of the first architecture was analyzed using Mosquitto metrics vs. AWS metrics. The results showed that the performance in the IoT–cloud with a fog layer was significantly better than without the fog layer, as the number of IoT devices and the number of subscribe and publish commands increased. The results also showed that the use of a Python script or fog-aided IoT–cloud environment resulted in the same performance. The results of our third analysis showed that as the number of IoT devices increased, the processability of the fog device in the fog-aided IoT–cloud architectures outperformed that of the AWS cloud. This study aimed to educate readers on different methods that can be used to implement IoT cloud environments and to compare the performance for each. It can also guide researchers by providing different ways to implement fog-aided IoT–cloud systems.

In the future, we plan to extend this study by using the same implementation frameworks presented in this paper to analyze the performance using different cloud providers, protocols, fog devices, and IoT devices. The presented implementation frameworks will also be used to investigate and address security issues in fog-aided IoT–cloud systems.

## Figures and Tables

**Table 1:** Summary of the equipment used in the two architectures.

Equipment Name	Equipment Type	Quantity	Purpose
DHT11	Temperature-Humidity Sensor	3	Generate Real Life Data
Raspberry Pi	Version 3 Model B	4	Enable WiFi and Provide Huge Processing Power and Storage
Micro SD Card	32GB ImageMate Plus 130 MB/s Read	4	Initial Storage for The Operating System and Files
Monitor	HP	4	Provide a Visual Display
Keyboard and Mouse	HP	4	Useful for Working on a Raspberry Pi
Power Supply/Adapter	CanaKit	4	Supply the Power for the Raspberry Pi
HDMI Cable	onn	4	Connect the Raspberry Pi to a Monitor

**Table 2:** AWS IoT message broker metrics for the North Virginia datacenter (cloud layer) using a bridge—first and second architectures.

AWS IoT Message Broker Metrics on N. Virginia Datacenter (Cloud Layer) Using Bridge—First Architecture						AWS IoT Message Broker Metrics on N. Virginia Datacenter (Cloud Layer)—Second Architecture					
Number of IoT Devices	1					Number of IoT Devices	1				
Number of subscribing and Publishing	2					Number of Subscribing and Publishing	2				
AWS IoT Metrics in Minutes (m)	0.5	1	5	15	60	AWS IoT Metrics in Minutes (m)	0.5	1	5	15	60
Connect Success	2	2	2	2	2	Connect Success	2	2	2	2	2
Ping Success	2	2	8	29	120	Ping Success	5	5	25	75	297
PublishIn Success	44	44	206	586	2360	PublishIn Success	60	60	300	900	3580
PublishOut Success	44	44	206	586	2360	PublishOut Success	60	60	300	900	3580
Subscribe Success	2	2	2	2	2	Subscribe Success	2	2	2	2	2
Unsubscribe Success	2	2	2	2	2	Unsubscribe Success	2	2	2	2	2
AWS IoT Message Broker Metrics on N. Virginia Datacenter (Cloud Layer) using Bridge—First Architecture						AWS IoT Message Broker Metrics on N. Virginia Datacenter (Cloud Layer)—Second Architecture					
Number of IoT Devices	2					Number of IoT Devices	2				
Number of Subscribing and Publishing	4					Number of Subscribing and Publishing	4				
AWS IoT Metrics in Minutes	0.5	1	5	15	60	AWS IoT Metrics in Minutes	0.5	1	5	15	60
Connect Success	4	4	4	4	4	Connect Success	6	6	35	111	432
Ping Success	2	2	8	29	120	Ping Success	1	1	8	20	85
PublishIn Success	70	70	350	1110	4590	PublishIn Success	62	62	322	965	3850
PublishOut Success	70	70	350	1110	4590	PublishOut Success	51	51	322	965	3850
Subscribe Success	4	4	4	4	4	Subscribe Success	6	6	35	111	432
Unsubscribe Success	4	4	4	4	4	Unsubscribe Success	4	4	4	4	4
AWS IoT Message Broker Metrics on N. Virginia Datacenter (Cloud Layer) using Bridge—First Architecture						AWS IoT Message Broker Metrics on N. Virginia Datacenter (Cloud Layer)—Second Architecture					
Number of IoT Devices	3					Number of IoT Devices	3				
Number of Subscribing and Publishing	6					Number of Subscribing and Publishing	6				
AWS IoT Metrics in Minutes	0.5	1	5	15	60	AWS IoT Metrics in Minutes	0.5	1	5	15	60
Connect Success	6	6	6	6	6	Connect Success	12	12	76	228	893
Ping Success	2	2	9	29	119	Ping Success	2	1	5	14	60
PublishIn Success	115	115	548	1660	6510	PublishIn Success	68	68	359	1050	4240
PublishOut Success	115	115	548	1660	6510	PublishOut Success	58	68	359	1050	4240
Subscribe Success	6	6	6	6	6	Subscribe Success	12	12	76	228	893
Unsubscribe Success	6	6	6	6	6	Unsubscribe Success	6	6	6	6	6

**Table 3:** AWS IoT message broker metrics on North Virginia datacenter (cloud layer) using a bridge vs. Python—first architecture.

AWS IoT Message Broker Metrics on N. Virginia Datacenter (Cloud Layer) Using Python Script—First Architecture						AWS IoT Message Broker Metrics on N. Virginia Datacenter (Cloud Layer) Using Bridge—First Architecture					
Number of IoT Devices	1					Number of IoT Devices	1				
Number of Subscribing and Publishing	2					Number of Subscribing and Publishing	2				
AWS IoT Metrics in Minutes	0.5	1	5	15	60	AWS IoT Metrics in Minutes	0.5	1	5	15	60
Connect Success	2	2	2	2	2	Connect Success	2	2	2	2	2
Ping Success	2	2	10	30	119	Ping Success	2	2	8	29	120
IoT PublishIn Success	46	46	206	590	2360	IoT PublishIn Success	44	44	206	586	2360
IoT PublishOut Success	46	46	206	590	2360	IoT PublishOut Success	44	44	206	586	2360
Subscribe Success	2	2	2	2	2	Subscribe Success	2	2	2	2	2
IoT Unsubscribe Success	2	2	2	2	2	IoT Unsubscribe Success	2	2	2	2	2
AWS IoT Message Broker Metrics on N. Virginia Datacenter (Cloud Layer) using Python Script—First Architecture						AWS IoT Message Broker Metrics on N. Virginia Datacenter (Cloud Layer) using Bridge—First Architecture					
Number of IoT Devices	2					Number of IoT Devices	2				
Number of Subscribing and Publishing	4					Number of Subscribing and Publishing	4				
AWS IoT Metrics in Minutes	0.5	1	5	15	60	AWS IoT Metrics in Minutes	0.5	1	5	15	60
Connect Success	4	4	4	4	4	Connect Success	4	4	4	4	4
Ping Success	2	2	9	29	118	Ping Success	2	2	8	29	120
IoT PublishIn Success	76	76	359	1170	4680	IoT PublishIn Success	70	70	350	1110	4590
IoT PublishOut Success	76	76	359	1170	4680	IoT PublishOut Success	70	70	350	1110	4590
Subscribe Success	4	4	4	4	4	Subscribe Success	4	4	4	4	4
IoT Unsubscribe Success	4	4	4	4	4	IoT Unsubscribe Success	4	4	4	4	4
AWS IoT Message Broker Metrics on N. Virginia Datacenter (Cloud Layer) using Python Script—First Architecture						AWS IoT Message Broker Metrics on N. Virginia Datacenter (Cloud Layer) using Bridge—First Architecture					
Number of IoT Devices	3					Number of IoT Devices	3				
Number of Subscribing and Publishing	6					Number of Subscribing and Publishing	6				
AWS IoT Metrics in Minutes	0.5	1	5	15	60	AWS IoT Metrics in Minutes	0.5	1	5	15	60
Connect Success	6	6	6	6	6	Connect Success	6	6	6	6	6
Ping Success	1	2	10	30	118	Ping Success	2	2	9	29	119
IoT PublishIn Success	117	117	534	1680	6650	IoT PublishIn Success	115	115	548	1660	6510
IoT PublishOut Success	117	117	534	1680	6650	IoT PublishOut Success	115	115	548	1660	6510
Subscribe Success	6	6	6	6	6	Subscribe Success	6	6	6	6	6
IoT Unsubscribe Success	6	6	6	6	6	IoT Unsubscribe Success	6	6	6	6	6

**Table 4:** Mosquitto message broker metrics on the fog layer using a bridge—first architecture.

Number of IoT Devices	One IoT Device						
Number of Subscribing and Publishing	Two Subscribing and Two Publishing						
Mosquitto Message Broker Metrics as $SYS Topics	30 (S)	1 (Min)	5 (Min)	15 (Min)	30 (Min)	45 (Min)	1 (H)
$SYS/Broker/Load/Messages/Received 1(m)	25.31	36.26	27.43	46.25	47.81	25.96	44.70
$SYS/Broker/Load/Messages/Sent 1(m)	137.87	177.35	179.52	215.36	199.56	188.81	213.35
$SYS/Broker/Load/Publish/Received 1(m)	22.68	33.07	23.95	43.88	45.38	23.43	42.69
$SYS/Broker/Load/Publish/Sent 1(m)	135.23	174.15	176.03	213.00	197.14	186.28	211.34
$SYS/Broker/Load/Bytes/Received 1(m)	802.93	1135.98	849.26	1478.98	1524.90	791.00	1439.38
$SYS/Broker/Load/Bytes/Sent 1(m)	5315.03	6898.23	7230.76	8637.79	8029.54	7694.46	8610.21
$SYS/Broker/Load/Sockets 1(m)	1.05	0.61	0.92	0.09	0.12	0.94	0.12
$SYS/Broker/Load/Connections 1(m)	1.05	0.61	0.92	0.09	0.12	0.94	0.12
$SYS/Broker/Subscriptions/Count	2	2	2	2	2	2	2
$SYS/Broker/Heap/Current	30,224	30,232	30,360	30,148	30,084	29,744	30,132
$SYS/Broker/Messages/Received	37	65	225	663	1285	1885	2515
$SYS/Broker/Messages/Sent	202	329	1144	3250	6356	9416	12,552
$SYS/Broker/Publish/Messages/Received	32	58	209	625	1213	1787	2375
$SYS/Broker/Publish/Messages/Sent	197	322	1128	3212	6284	9310	12,412
$SYS/Broker/Bytes/Received	1154	2029	7149	21,219	41,165	60374	80,633
$SYS/Broker/Bytes/Sent	7730	12,711	45,329	129,895	255,029	378,636	505,171
$SYS/Broker/Publish/Bytes/Received	160	290	1045	3125	6065	8935	11,875
$SYS/Broker/Publish/Bytes/Sent	806	1400	5505	16,551	33,192	49,748	66,818

**Table 5:** Mosquitto message broker metrics on the fog layer using a bridge—first architecture.

Number of IoT Devices	Two IoT Devices						
Number of Subscribing and Publishing	Four Subscribing and Four Publishing						
Mosquitto Message Broker Metrics as $SYS Topics	30 (S)	1 (Min)	5 (Min)	15 (Min)	30 (Min)	45 (Min)	1 (H)
$SYS/Broker/Load/Messages/Received 1(m)	59.48	79.02	109.81	119.86	109.03	123.12	114.70
$SYS/Broker/Load/Messages/Sent 1(m)	175.38	226.02	296.83	312.57	296.21	307.21	298.85
$SYS/Broker/Load/Publish/Received 1(m)	38.21	53.18	73.62	79.78	74.34	82.05	78.49
$SYS/Broker/Load/Publish/Sent 1(m)	154.11	200.18	260.63	272.49	261.52	266.13	262.63
$SYS/Broker/Load/Bytes/Received 1(m)	2124.60	2767.93	3916.20	4291.54	3854.92	4393.78	4074.59
$SYS/Broker/Load/Bytes/Sent 1(m)	5981.40	7807.80	10,344.23	10,803.61	10,424.09	10,568.27	10,457.24
$SYS/Broker/Load/Sockets 1(m)	19.69	23.40	33.62	37.64	32.03	38.43	34.27
$SYS/Broker/Load/Connections 1(m)	19.69	23.40	33.64	37.65	32.12	38.43	34.27
$SYS/Broker/Subscriptions/Count	4	4	4	4	4	4	4
$SYS/Broker/Heap/Current	30,268	30,268	30,252	30,268	30,252	30,260	30,204
$SYS/Broker/Messages/Received	84	143	593	1747	3433	5194	6954
$SYS/Broker/Messages/Sent	253	416	1632	4686	9154	13,704	18,242
$SYS/Broker/Publish/Messages/Received	53	94	398	1157	2283	3451	4618
$SYS/Broker/Publish/Messages/Sent	222	367	1437	4096	8004	11961	15906
$SYS/Broker/Bytes/Received	2976	5015	21,111	62,781	123,353	186,822	250,274
$SYS/Broker/Bytes/Sent	8609	14,285	56,570	162,328	318,376	476,257	633,619
$SYS/Broker/Publish/Bytes/Received	265	470	1990	5785	11,415	17,255	23,090
$SYS/Broker/Publish/Bytes/Sent	942	1650	7159	21,442	42,979	65,025	87076

**Table 6:** Mosquitto message broker metrics on the fog layer using a bridge—first architecture.

Number of Iot Devices	Three IoT Devices						
Number of Subscribing and Publishing	Six Subscribing and Six Publishing						
Mosquitto Message Broker Metrics as $SYS Topics	30 (S)	1 (Min)	5 (Min)	15 (Min)	30 (Min)	45 (Min)	1 (H)
$SYS/Broker/Load/Messages/Received 1(m)	90.09	129.50	172.49	163.15	163.81	172.50	179.13
$SYS/Broker/Load/Messages/Sent 1(m)	207.44	279.24	359.50	357.54	353.60	352.41	369.72
$SYS/Broker/Load/Publish/Received 1(m)	55.43	81.41	109.62	99.23	98.21	107.07	115.52
$SYS/Broker/Load/Publish/Sent 1(m)	172.78	231.14	296.63	293.61	287.99	286.97	306.10
$SYS/Broker/Load/Bytes/Received 1(m)	3329.39	4744.07	6384.79	6124.10	6170.40	6480.58	6627.83
$SYS/Broker/Load/Bytes/Sent 1(m)	6672.01	8948.99	11,686.44	11,697.09	11,528.00	11,394.30	12,088.13
$SYS/Broker/Load/Sockets 1(m)	33.08	45.51	60.27	61.48	63.11	63.81	61.52
$SYS/Broker/Load/Connections 1(m)	33.08	45.51	60.29	61.49	63.11	63.82	61.63
$SYS/Broker/Subscriptions/Count	6	6	6	6	6	6	6
$SYS/Broker/Heap/Current	30,376	30,320	30,268	30,312	30,376	30,240	30,252
$SYS/Broker/Messages/Received	129	228	951	2689	5310	7915	10,503
$SYS/Broker/Messages/Sent	299	505	1992	5639	11,077	16,495	21,823
$SYS/Broker/Publish/Messages/Received	79	142	603	1699	3333	4973	6612
$SYS/Broker/Publish/Messages/Sent	249	419	1644	4649	9100	13,553	17,932
$SYS/Broker/Bytes/Received	4736	8349	35210	100,003	197,975	295,130	391,395
$SYS/Broker/Bytes/Sent	9603	16202	64301	183,194	359,880	536,549	710,168
$SYS/Broker/Publish/Bytes/Received	395	710	3015	8495	16,665	24,865	33,060
$SYS/Broker/Publish/Bytes/Sent	1082	1920	8315	24,546	49,094	73,862	98,359

## Data Availability

Not applicable.
